# Threshold-Anchored Mechanomyography Metrics for Patient Stratification in Spinal Decompression: Associations with Early Pain Outcomes

**DOI:** 10.3390/jpm15120564

**Published:** 2025-11-21

**Authors:** Muwaffak Abdulhak, Ross Jones, David Nay, Christopher Wybo

**Affiliations:** 1 Department of Neurosurgery, Henry Ford Hospital, 2799 W Grand Blvd, Detroit, MI 48202, USA; 2 Flagstaff Bone and Joint, 525 North Switzer Canyon Drive, Flagstaff, AZ 86001, USA; 3 Neuralytix, LLC., 138 S Park Square, Suite 203, Fruita, CO 81521, USA

**Keywords:** MMG, mechanomyography, spinal decompression, intraoperative nerve health assessment, pilot study, threshold-anchored metrics, intraoperative decision-making, decompression biomarkers, personalized medicine, patient stratification

## Abstract

**Background/Objectives:** Spinal decompression surgery shows variable outcomes, with reoperation rates up to 37.5%. Surgeons lack objective intraoperative tools to assess decompression adequacy. Mechanomyography (MMG) measures nerve excitability through mechanical muscle responses to electrical stimulation. While compressed nerves require higher stimulation thresholds, optimal quantification approaches remain undefined. We explored associations between intraoperative MMG threshold changes and six-week pain outcomes, comparing metrics anchored to a 2.0 mA reference threshold versus percentage-based measures. **Methods:** Prospective exploratory pilot study of 42 patients (112 nerves) undergoing lumbar or cervical decompression. MMG thresholds were recorded pre- and post-decompression. Numeric Pain Scale scores were obtained preoperatively and at six weeks. Three metrics were compared: percentage change, Threshold Reduction Ratio (TRR; measuring proportion of threshold elevation above 2.0 mA eliminated by decompression), and Threshold Excess (TE; residual threshold remaining above 2.0 mA), with TRR and TE anchored to 2.0 mA based on published normal ranges. **Results:** Among 40 patients with baseline pain, threshold-anchored metrics showed substantially stronger correlations with pain improvement than percentage-based measures (TRR: r = 0.656, *p* < 0.001 vs. percentage: r = 0.397, *p* = 0.011). Threshold Excess was associated with a linear dose–response: each 1 mA above 2.0 mA corresponded to 6.3% less pain improvement (*p* = 0.001). Patients achieving ≤2.0 mA had 6.1-fold increased odds of complete pain relief versus those above 2.0 mA (76.5% vs. 34.8%, *p* = 0.013). Internal leave-one-out cross-validation suggested internal stability (TRR shrinkage ≈ 9.3%; TE’s dose–response slope remained stable). **Conclusions:** In this exploratory pilot study, threshold-anchored MMG metrics (TRR and TE) showed stronger correlations with early pain outcomes than percentage-based measures. These exploratory findings require external validation in independent cohorts before clinical implementation. If validated prospectively, these metrics could provide objective, real-time feedback for clinical interpretation to inform surgical decision-making during spinal decompression, enabling surgeons to tailor decompression to individual physiology rather than relying on standardized anatomical criteria. Future work should explore patient-specific threshold targets that account for age, chronicity, and comorbidities.

## 1. Introduction

Spinal decompression surgery aims to relieve nerve root compression and improve pain and function, yet outcomes remain variable. A meta-analysis of decompression-only procedures for degenerative spondylolisthesis reported an overall reoperation rate of 9.1% (95% CI 6.5–11.7%), increasing to 11.7% by 3–5 years [1]. In routine practice, long-term reoperation remains substantial even in modern surgical cohorts: a 2022 prospective analysis of lumbar decompression identified a cumulative revision rate of 11.3% at three years, and those requiring reoperation had significantly worse outcomes at final follow-up [2]. Economically, the burden is considerable: back pain carries among the highest indirect costs in the U.S. workforce [3], and the economic burden continues to rise: recent analyses show that procedural costs for spinal deformity surgery have increased substantially while payments have lagged, creating a financial imperative to prevent revision procedures [4]. Complication-related care also compounds expense; five-year modeling estimates ≈ $35,000 in added costs per patient following spinopelvic complications [5]. Together, these procedure-level expenses occur within a system that spends about $134.5 billion annually on low back and neck pain [3,6]. Clinically, Failed Back Surgery Syndrome (FBSS)—also termed Persistent Spinal Pain Syndrome Type 2 in contemporary nomenclature—describes persistent or recurrent pain despite anatomically successful surgery—and affects a substantial proportion of lumbar surgery patients [7], underscoring the need for objective intraoperative tools to help confirm decompression adequacy.

A fundamental challenge underlying these outcomes is the absence of objective intraoperative tools for assessing nerve decompression adequacy. Surgeons traditionally rely on visual inspection, tactile feedback, and preoperative imaging to determine when sufficient decompression has been achieved. However, systematic reviews demonstrate significant limitations in this subjective approach. Intraoperative imaging modalities such as ultrasound and CT frequently reveal residual compression that visual assessment missed, often requiring additional bone removal [8].

Current intraoperative neurophysiological monitoring techniques have recognized limitations for assessing decompression adequacy. Meta-analyses report that somatosensory evoked potentials (SSEPs) demonstrate high specificity (95–97%) but modest sensitivity (44–46%) for detecting neural compromise [9,10]. Similarly, motor-evoked potentials show high accuracy but primarily detect injury rather than confirm adequacy. Some patients develop new postoperative deficits despite monitoring [11], highlighting that current techniques primarily detect injury rather than confirm adequacy. This creates an important asymmetry: while monitoring effectively indicates when surgeons have decompressed too aggressively (injury detection), it provides limited guidance on whether decompression is sufficient (adequacy confirmation).

Intraoperative imaging can identify residual compression, with contemporary reviews confirming that three-dimensional imaging reveals the need for plan modification in ~20% of cases [12]. Even in technically demanding ossification of the posterior longitudinal ligament (OPLL), mobile intraoperative CT during the floating method has been shown to reduce postoperative insufficient decompression [13]. Recent evidence with modern cone-beam CT demonstrates that even with three-dimensional guidance, intraoperative imaging prompts immediate revision in a substantial proportion of cases—for instance, requiring pedicle screw repositioning in 17.5% of dorsal instrumentation procedures—confirming that initial visual assessment frequently misses critical details [14]. Despite these potential insights, CT-based approaches carry radiation exposure risks to patients and staff while adding time and complexity to the procedure.

Beyond CT-based modalities, real-time intraoperative ultrasound has been piloted in traumatic cervical spinal cord injury to assess adequacy immediately after laminectomy and to guide additional decompression when residual compression is detected [15]. However, while navigation and robotic systems enhance anatomical precision for instrumentation, they remain focused on bony anatomy and do not provide functional assessment of nerve decompression adequacy [16,17].

Mechanomyography (MMG) represents a novel approach to intraoperative nerve health assessment that measures mechanical muscle responses to electrical nerve stimulation through accelerometer sensors placed on the skin surface about target muscles [18]. Unlike electromyography (EMG), which detects electrical activity, MMG captures the actual mechanical contraction of muscle fibers, providing a quantifiable response with a strong signal-to-noise ratio. Contemporary reviews of stimulation-based MMG further detail sensor implementations and signal interpretation, supporting its use as a practical, low-frequency mechanical complement to EMG in the operative setting [19].

The technology has shown promise in peripheral nerve surgery, where Guerrero et al. demonstrated that 80% of patients had detectable MMG signals with significant threshold improvements following decompression (mean reduction from 1.59 ± 0.19 mA to 0.47 ± 0.03 mA, *p* = 0.0002) [20] and Javeed and colleagues showed that MMG thresholds correlated with compound muscle action potentials (in peroneal nerves) and clinical outcomes (PROMIS Physical Function, Oswestry Disability Index) at final follow-up (*p* < 0.05) [21]. In spine surgery, Zakaria and colleagues showed that MMG could detect pedicle screw breaches with 99.5% negative predictive value using a threshold cutoff of 12 mA (sensitivity 80.4%, specificity 80.6%) in 122 patients with 890 screws [22].

Prior work has established that chronically compressed nerve roots require higher electrical stimulus intensities for activation [23], and that surgical decompression typically reduces these thresholds [24]. Wessell et al. demonstrated that 90% of patients had elevated pre-decompression thresholds (≥2.0 mA) and 98% showed threshold reduction ≥1 mA following decompression, with mean post-decompression values of 2.04 ± 2.22 mA and concurrent VAS pain improvement from 6.8 to 1.1 [24]. While this established that threshold reduction correlates with pain improvement (*p* < 0.001), the strength, functional form, and clinical implications of this relationship remained unquantified. Sommer et al. recently reported mean stimulation threshold reductions of 3.4 mA for cervical foraminotomies and 4.4 mA for far lateral discectomies, with smaller changes during minimally invasive transforaminal lumbar interbody fusion (MIS-TLIF), where a true pre-decompression baseline is difficult to obtain [25]. However, these studies did not quantify the magnitude of the threshold-outcome relationship or identify specific post-decompression threshold targets—critical gaps for surgical decision-making.

A fundamental question in quantifying nerve recovery is whether percentage improvement from baseline provides optimal outcome prediction. Percentage-based metrics treat all baseline values as equivalent: a nerve stimulation threshold reducing from 15 mA to 10 mA (33% improvement) receives the same score as one normalizing from 3 mA to 2 mA (also 33% improvement) However, these outcomes may be clinically divergent—the former remains markedly elevated above physiological ranges while the latter achieves normalization. If post-decompression proximity to normal thresholds predicts outcomes better than arbitrary percentage change, metrics anchored to physiological reference values might outperform percentage-based approaches. This hypothesis has not been formally tested.

Current surgical practice applies relatively uniform decompression protocols across patients based on anatomical criteria and visual inspection. However, nerve pathology, chronicity, and recovery potential vary substantially between individuals. A patient with acute compression may achieve rapid threshold normalization with minimal decompression, while another with chronic compression might require more extensive intervention to achieve similar physiologic recovery—if achievable at all given irreversible changes. Personalized spine surgery requires objective tools to tailor intervention extent to individual nerve physiology in real-time, optimizing outcomes while minimizing procedural risk. Such individualized approaches could reduce both under-decompression (leading to persistent symptoms) and over-decompression (increasing complication risk) by providing patient-specific physiologic feedback.

We conducted this exploratory pilot study to: (1) quantify associations between MMG threshold changes and six-week pain outcomes, (2) explore different quantification approaches—including metrics anchored to a clinically referenced threshold—to determine which might best predict outcomes, (3) characterize dose–response relationships between post-decompression threshold values and clinical outcomes, and (4) establish feasibility and effect size estimates for future randomized trials.

## 2. Materials and Methods

### 2.1. Study Design and Setting

We conducted a prospective observational cohort study at a single academic medical center between January and December 2023. The Henry Ford Health System Institutional Review Board (Detroit, MI, USA) approved the protocol (IRB #14745). All participants provided written informed consent. The study followed STROBE reporting guidelines [26].

### 2.2. Participants

*Inclusion criteria:* Adults aged 18–70 years with clinical and radiographic nerve root compression requiring surgical decompression, scheduled for lumbar or cervical spine surgery involving at least one nerve root, and ability to complete 6-week follow-up.

*Exclusion criteria:* Advanced peripheral neuropathy, diabetes, previous surgery at index level, significant psychological disturbance, or pregnancy.

### 2.3. Sample Size Determination

Based on pilot study methodology guidelines [27], a sample size of 50 patients was determined to provide adequate precision for estimating correlation coefficients. This aligns with empirical pilot study sizes documented in the literature [28] and provides 80% power to detect r ≥ 0.40 at α = 0.05 [28].

### 2.4. Mechanomyography Protocol

We used the DePuy Synthes Sentio MMG system (FDA-cleared for nerve locating, mapping, and neurophysiologic assessment) (DePuy Synthes Spine, Raynham, MA, USA). Accelerometer sensors were placed on muscles corresponding to the relevant nerve roots based on standard anatomical references [29].

- *Cervical:* Trapezius (C3–C4), deltoid/biceps (C5–C6), triceps/adductor pollicis (C6–C8)
- *Lumbar:* Vastus medialis (L2–L4), tibialis anterior (L4–L5), biceps femoris (L5–S2), gastrocnemius (S1–S2)

Following exposure but prior to decompression, the probe was placed in direct contact with the exposed nerve root. Stimulation (0.1 ms pulse width, 4 Hz) began at 1 mA and was increased in 1 mA increments until an MMG response was detected. The lowest stimulation current (mA) producing a detectable MMG response was recorded as the pre-decompression threshold. Measurements were repeated after the surgeon deemed decompression complete and recorded as the post-decompression threshold.

### 2.5. Surgical Technique

Intraoperatively, surgical access and exposure of the nerves followed standard clinical practice. Decompression extent was based on preoperative imaging and intraoperative findings, consistent with contemporary standards [30]. Adequacy was determined by visual inspection, palpation, and nerve mobility.

### 2.6. Anesthesia Protocol

General anesthesia with neuromuscular blockade limited to intubation. Train-of-four (TOF) monitoring, which delivers four sequential supramaximal electrical stimuli to the ulnar nerve to quantify neuromuscular junction recovery, confirmed adequate paralytic reversal (TOF ratio ≥ 0.9, indicating recovery of at least 90% of baseline neuromuscular transmission) before MMG testing [31].

### 2.7. Outcome Measures

*Primary Outcome:* Change in Numeric Pain Scale (NPS) score for radicular pain from baseline to 6 weeks postoperatively [32].

*Secondary Outcomes:* 1. Proportion achieving minimal clinically important difference (MCID) on the NPS, pre-specified as ≥2-point reduction or ≥30% improvement from baseline based on contemporary spine-specific literature [33,34] 2. Association between novel MMG metrics and pain improvement 3. Diagnostic accuracy of MMG for predicting clinical improvement.

### 2.8. Statistical Analysis

#### 2.8.1. Analytical Approach and Rationale for Exploratory Metric Development

This exploratory pilot study followed IDEAL Stage 2b/3 framework for surgical innovation in which hypothesis generation and metric development are primary objectives [35]. The original IRB-approved protocol specified prospective data collection with the observation that normal nerve thresholds fall within 1–2 mA based on published MMG literature [24] but included no pre-planned statistical analyses. The protocol’s intent was to characterize MMG-outcome associations and establish feasibility for future controlled trials.

During manuscript development, we conducted statistical analyses to quantify observed associations and explore optimal quantification approaches. The 1–2 mA range was protocol-specified based on published evidence [24]. We selected 2.0 mA—the upper boundary of this pre-specified range—as the operational reference threshold (not data-driven), as stimulation thresholds < 2.0 mA represent normal nerve function [24]. However, the specific metric formulations (TRR and TE) were developed iteratively during analysis to test whether measuring distance from this physiological reference might predict outcomes better than percentage-based approaches.

This exploratory metric development is appropriate for pilot studies, where generating candidate metrics for prospective testing is a legitimate scientific objective [27]. To assess whether observed associations represented genuine signal versus overfitting, we performed rigorous internal cross-validation (Section 2.8.4). The biological rationale for threshold-anchored metrics—that proximity to physiological normal values may predict outcomes better than arbitrary percentage improvements—is independent of the observed data and grounded in fundamental neurophysiology.

Given the hypothesis-generating nature of this work, we did not correct for multiple comparisons. All findings should be interpreted as preliminary effect-size estimates requiring external validation in independent cohorts before clinical application. The primary contribution of this work is not definitive validation of specific numerical cutpoints, but rather the conceptual framework and feasibility demonstration to enable adequately powered prospective studies.

Primary analysis examined the relationship between MMG threshold changes and pain improvement among patients with baseline pain > 0 (*n* = 40).

#### 2.8.2. Development of Threshold-Anchored Metrics:

We employed an iterative approach to identify metrics optimally capturing the MMG-outcome relationship. Initial analyses examined percentage threshold reduction. To explore whether measuring distance from a reference threshold might improve predictive value, we developed two candidate metrics:

1. **Threshold Reduction Ratio (TRR):** Quantifies the proportion of baseline threshold elevation (above 2.0 mA) eliminated by decompression. For each nerve with baseline ≥ 2 mA:
  - Baseline elevation = MMG_pre − 2.0 mA;
  - Post-decompression elevation = max(MMG_post − 2.0 mA, 0);
  - TRR = (Baseline elevation − Post-decompression elevation)/Baseline elevation;
  - Patient-level TRR = mean across nerves with baseline elevation.
2. **Threshold Excess (TE):** Quantifies post-decompression threshold remaining above 2.0 mA:
  - TE = max (mean post-MMG − 2.0 mA, 0);
  - Provides an absolute operational benchmark for residual pathology.

#### 2.8.3. Selection of 2.0 mA Reference Threshold:

The study protocol pre-specified 1–2 mA as the normal nerve range based on published literature [24]. We selected 2.0 mA as the operational reference threshold, representing the upper boundary of this protocol-specified range and recognizing the resolution limits of the Sentio system. This selection aligns with Wessell et al. [24] who noted that stimulation thresholds < 2.0 mA would be considered “normal” and reported mean post-decompression values of 2.04 ± 2.22 mA in successfully decompressed nerves.

TRR and TE formulations were developed iteratively during analysis to explore whether measuring distance from this reference improved outcome prediction compared to percentage-based metrics. Given the hypothesis-generating nature of this work, we did not correct for multiple comparisons.

#### 2.8.4. Internal Cross-Validation

To address the concern that exploratory metric development might yield inflated performance estimates, we performed rigorous leave-one-out cross-validation (LOOCV) for all three quantification metrics among the 40 patients with baseline pain > 0. For each patient, we trained linear regression models relating MMG metrics to percentage pain improvement using data from the remaining 39 patients, then predicted the outcome for the held-out patient. This process was repeated 40 times, providing an unbiased estimate of predictive performance in new patients. Cross-validated correlations represent correlations between actual outcomes and predictions from held-out test sets. Shrinkage was calculated as the percentage decrease from apparent (full-data) to cross-validated performance; values < 20% indicate robust generalization.

### 2.9. Use of Generative AI

This study employed generative AI as a computational and editorial tool, with rigorous human oversight and multi-system verification at all stages. Statistical Analysis: The research team designed and conducted all primary statistical analyses, including test selection and result interpretation. Generative AI performed computational tasks such as confidence interval calculations, z-score computations, cross-validation, and robustness checks. All results were independently verified by the authors and cross-validated across multiple AI systems for convergence and reproducibility. (reproducible analysis code is provided in Supplemental Materials File S3) Literature Review: AI-assisted searches identified relevant publications. All citations were independently verified by the authors for accuracy and relevance. Manuscript Preparation: AI tools generated portions of the initial draft, which underwent substantial revision, reorganization, and verification by all authors. Multiple AI systems reviewed drafts for methodological weaknesses, statistical errors, and logical inconsistencies, with issues addressed by the team. All scientific content, methodological decisions, interpretations, and conclusions represent the authors’ independent judgment. The authors take full responsibility for the work’s accuracy, validity, and integrity.

## 3. Results

### 3.1. Cohort Characteristics

Of 50 enrolled patients, 42 (84%) completed 6-week follow-up. The cohort included 112 nerve decompressions across 42 patients (Table 1; see Supplemental Materials File S1 for patient level data and File S2 for aggregated summary tables). Two patients with baseline NPS = 0 underwent surgery for functional impairment rather than pain and were excluded from pain-outcome analyses, leaving 40 patients for correlation analyses. This exclusion was determined during analysis (not pre-specified) as percentage pain improvement is undefined when baseline = 0.

### 3.2. Intraoperative MMG Threshold Changes

#### 3.2.1. Nerve-Level Analysis

**Figure 1:**
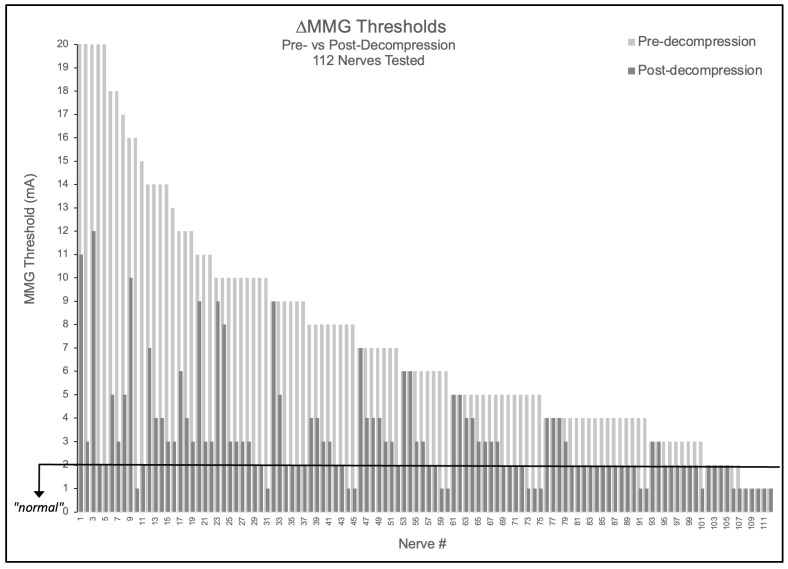
Nerve-level MMG threshold changes pre- versus post-decompression. Bar graph showing all 112 nerves tested, with light gray bars indicating pre-decompression thresholds and adjacent dark gray bars showing respective post-decompression thresholds. Black horizontal line indicates 2.0 mA normal threshold.

Of 112 nerves tested, 90.2% (101/112) demonstrated elevated baseline thresholds > 2.0 mA (mean 7.43 ± 4.85 mA). Following decompression, 89.1% (90/101) of elevated nerves improved by ≥1 mA, with 52.2% (47/90) of these improving nerves achieving complete normalization to ≤2.0 mA. Notably, no nerve demonstrated threshold worsening (0/112), suggesting the measurement technique did not cause detectable harm in this cohort (Figure 1). The mean threshold reduction was 4.34 ± 4.26 mA (48.7% improvement from baseline, *p* < 0.001).

#### 3.2.2. Patient-Level Analysis:

When averaging multiple nerves per patient, 95.2% (40/42) had elevated mean baseline thresholds > 2.0 mA (mean 8.5 ± 4.3 mA). Post-decompression, 98% (39/40) of patients with elevated baselines showed improvement ≥ 1 mA. The mean post-decompression threshold decreased to 3.4 ± 2.2 mA, representing a 5.2 ± 3.7 mA reduction (61% improvement, *p* < 0.001).

**Figure 2:**
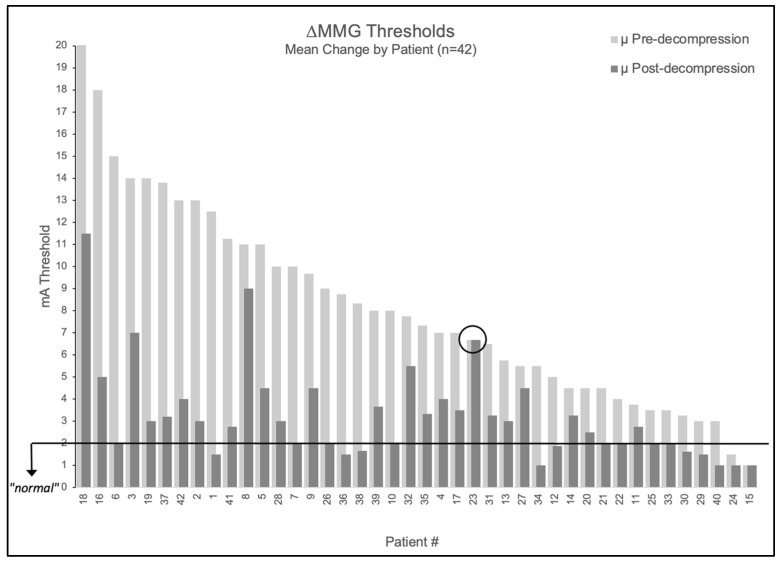
Patient-level MMG threshold changes pre- versus post-decompression. Bar graph showing all 42 patients tested, with light gray bars indicating pre-decompression thresholds and adjacent dark gray bars showing respective post-decompression thresholds. Black horizontal line indicates 2.0 mA normal threshold. Patient 23 is circled as the only patient to not experience a change in stimulation threshold following decompression.

**Figure 3:**
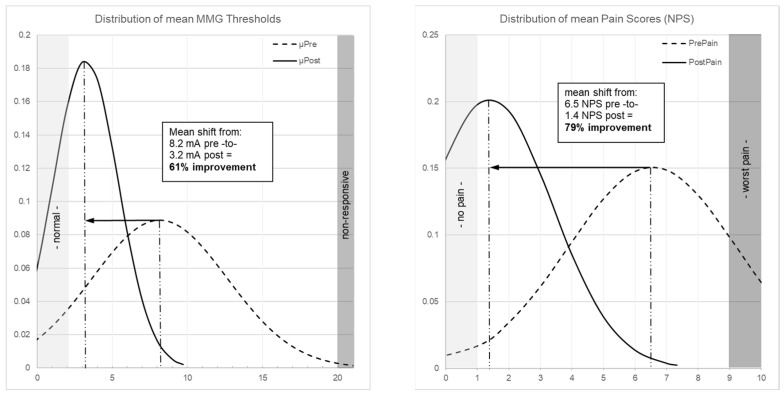
(**Left**) Patient-level distribution curves of MMG thresholds showing shift from mean 8.2 mA (dashed curve, pre-decompression) to 3.2 mA (solid curve, post-decompression), representing 61% improvement (*p* < 0.001). (**Right**) Distribution curve of pain scores showing shift from mean 6.5 NPS (dashed curve, pre-operative) to 1.4 NPS (solid curve, 6-week post-operative), representing 79% improvement (*p* < 0.001).

Figure 2 illustrates patient-level threshold changes. The population-level impact is shown through distribution curves: MMG thresholds shifted from a broad, right-skewed distribution centered at 8.2 mA to a narrower distribution centered at 3.2 mA (Figure 3 left), while pain scores shifted from 6.5 NPS pre-operatively to 1.4 NPS post-operatively (79% improvement, *p* < 0.001; Figure 3 right).

### 3.3. Pain Outcomes

At 6 weeks, 92.9% (39/42) of patients reported pain improvement, with 90.5% (38/42) achieving minimal clinically important difference (MCID) on the NPS (≥2-point reduction or ≥30% improvement). Mean radicular pain decreased from 6.8 ± 2.4 to 1.4 ± 2.0 (*p* < 0.001). Complete pain relief (NPS = 0) occurred in 54.8% (23/42) of patients.

**Figure 4:**
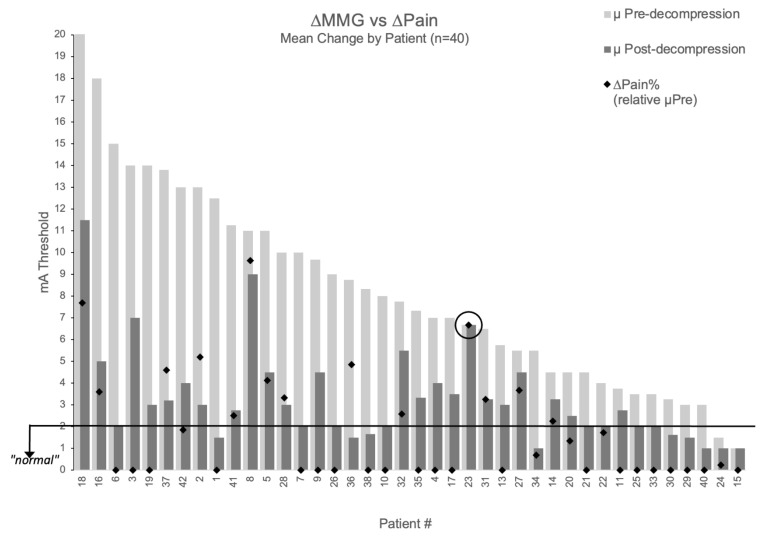
Relationship between MMG threshold changes and pain improvement. Patient-level visualization showing percentage pain improvement (black diamonds) overlaid on MMG threshold changes (light gray bars = pre-decompression, dark gray bars = post-decompression) for 40 patients with baseline pain > 0. Patients #39 and #12 excluded (baseline NPS = 0). Patient #23 circled showing concordant non-improvement in both MMG and pain.

The relationship between MMG improvement and pain relief is visualized in Figure 4, which displays percentage pain improvement overlaid on each patient’s threshold change (excluding two patients with baseline NPS = 0 where percentage improvement is undefined). One patient demonstrated perfect concordance between measurements and outcomes: despite surgical intervention deemed adequate by visual inspection, all three decompressed nerve roots maintained unchanged thresholds (9→9 mA, 6→6 mA, 5→5 mA; mean 6.67 mA), and radicular pain remained unchanged (NPS = 6 at both timepoints). This patient represented the only case (1/42, 2.4%) showing concordant non-improvement in both domains, suggesting MMG may provide objective information not available through traditional surgical assessment.

### 3.4. Comparative Performance of MMG Quantification Approaches

*Primary Analysis: Correlation with Pain Outcomes:* To determine the optimal approach for quantifying decompression, we compared three metrics in their ability to predict pain outcomes among patients with baseline pain (*n* = 40, excluding 2 patients with preoperative NPS = 0).

Threshold-anchored metrics showed substantially stronger correlations with percentage pain improvement than percentage-based measures in this cohort:

- % MMG change: r = 0.397 (95% CI 0.111–0.625, *p* = 0.011)
- Threshold Reduction Ratio (TRR): r = 0.656 (95% CI 0.426–0.807, *p* < 0.001)
- Threshold Excess (TE): r = −0.500 (95% CI −0.702 to −0.223, *p* = 0.001)

TRR showed a stronger correlation with pain outcomes than the percentage metric when evaluated on the same patients (matched subset, *n* = 38: r = 0.656 vs. r = 0.494). Steiger’s test for dependent correlations showed this difference was significant (Z = 2.09, two-sided *p* = 0.037). On the full cohort (*n* = 40), the percentage metric’s correlation was r = 0.397; however two patients in that full set could not contribute to TRR by definition (baseline ≤ 2.0 mA), so all inferential comparisons are reported on the matched subset for parity. In variance terms, on the matched subset TRR explains 43.1% of outcome variance versus 24.4% for the percentage-based metric.

These analyses combine lumbar and cervical decompressions to maximize sample size for this exploratory pilot study. Procedure-specific associations and optimal threshold values may differ and should be investigated in future studies with larger cohorts.

### 3.5. Threshold Reduction Ratio Analysis

**Figure 5:**
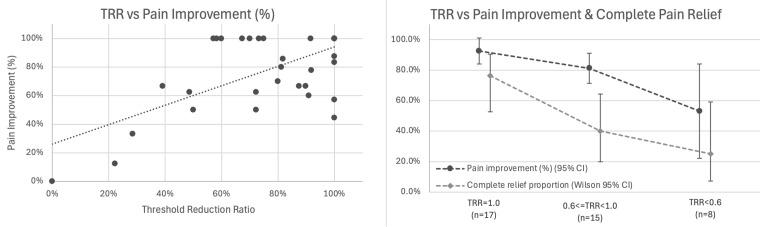
Threshold Reduction Ratio (TRR) correlates with pain outcomes. (**Left**) Scatter plot showing association between TRR and percentage pain improvement (r = 0.656, 95% CI 0.426–0.807, *p* < 0.001, *n* = 38). Trend line with 95% confidence interval shown. (**Right**) Stratified outcomes by TRR category showing mean percentage pain improvement (dark line with 95% CI) and proportion achieving complete pain relief (light line with Wilson 95% CI) for TRR = 1.0 (*n* = 17), 0.6 ≤ TRR < 1.0 (*n* = 15), and TRR < 0.6 (*n* = 8).

*Primary Analysis: Correlation with Pain Outcomes:* Among 38 patients with baseline pain > 0 and calculable TRR (two patients had baseline thresholds ≤ 2.0 mA), TRR showed the strongest association with percentage pain improvement (r = 0.656, 95% CI 0.426–0.807, *p* < 0.001; Figure 5 left).

*Exploratory Analysis: Stratification by TRR Category:* We examined outcomes across TRR tertiles (Figure 5 right, Table 2):

A dose–response gradient was observed in this cohort: higher TRR values (indicating greater elimination of baseline elevation) were associated with superior outcomes. In adjusted analysis controlling for baseline pain, number of nerves, and anatomical region, TRR remained the strongest predictor (β = 0.679, SE 0.130, *p* < 0.001), accounting for 43.1% of variance in pain improvement. Given the small subgroup sizes in this exploratory stratification (*n* = 8–17 per category), these findings provide preliminary evidence of dose–response gradients that require confirmation in larger cohorts.

### 3.6. Threshold Excess Analysis

**Figure 6:**
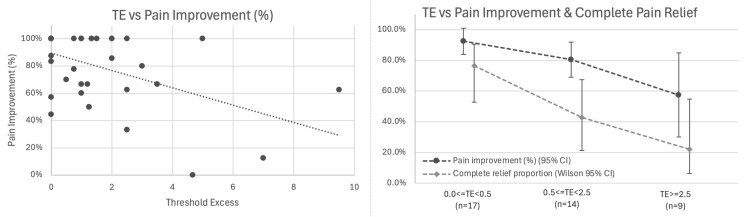
Threshold Excess (TE) inversely correlates with pain outcomes. (**Left**) Scatter plot showing inverse association between TE and percentage pain improvement (r = −0.500, 95% CI −0.702 to −0.223, *p* = 0.001, *n* = 40). Linear relationship: each 1 mA of TE associated with 6.3% less pain improvement. (**Right**) Stratified outcomes by TE category showing mean percentage pain improvement (dark line with 95% CI) and proportion achieving complete pain relief (light line with Wilson 95% CI) for 0.0 ≤ TE < 0.5 mA (*n* = 17), 0.5 ≤ TE < 2.5 mA (*n* = 14), and TE ≥ 2.5 mA (*n* = 9).

*Primary Analysis: Correlation with Pain Outcomes:* Among all 40 patients with baseline pain, TE showed inverse association with pain improvement: higher residual threshold elevation was associated with worse outcomes (r = −0.500, 95% CI −0.702 to −0.223, *p* = 0.001; Figure 6 left).

A linear relationship was observed in which each 1 mA of threshold excess was associated with 6.3% less pain improvement (β = −0.063, 95% CI −0.099 to −0.027, *p* = 0.001). In logistic regression for complete pain relief, each additional 1 mA of TE was associated with ~40% lower odds of complete relief (OR = 0.60; Wald 95% CI 0.36–0.98; bootstrap CI 0.21–0.87; *p* = 0.011), with predicted probabilities declining from ~68% at TE = 0 to ~6% at TE = 7 mA.

*Exploratory Analysis: Stratification by TE Category:* Stratified analysis by TE tertiles demonstrated this gradient (Figure 6 right, Table 3):

These exploratory stratified analyses, limited by small subgroup sizes, suggest threshold-based patient stratification may be feasible but require validation in adequately powered studies.

Notably, among 17 patients achieving TE = 0 (post-decompression ≤ 2.0 mA), further reduction below 2.0 mA showed no additional association with pain improvement (r = 0.094, *p* = 0.719), suggesting 2.0 mA may represent a functional sufficiency plateau beyond which additional threshold reduction provides diminishing benefit.

### 3.7. Relationship Between TRR and TE

While TRR and TE are associated (r = −0.756), they may capture distinct aspects of decompression:

- For continuous outcomes (magnitude of pain improvement): TRR alone R^2^ = 0.431; adding TE provided no additional explanatory power;
- For binary outcomes (complete relief): TE performed slightly better (AIC 50.15 vs. 50.77).

This pattern is consistent with TRR normalizing for baseline severity (relevant to improvement magnitude) while TE provides an absolute post-operative benchmark (relevant to categorical outcomes).

### 3.8. Internal Cross-Validation Analysis

Leave-one-out cross-validation revealed substantial differences in generalizability across metrics (Table 4). TRR demonstrated minimal shrinkage (9.3%), with cross-validated correlation remaining strong (r = 0.597) and retaining 82% of explained variance (cross-validated R^2^ = 35.6%). In contrast, percentage-based %MMG showed substantial shrinkage (49.3%), with cross-validated correlation declining to r = 0.201 and near-complete collapse of explained variance (cross-validated R^2^ = 4.0%, representing 75% loss from apparent R^2^ = 15.7%). These findings suggest TRR showed internal stability, while percentage-based metrics provide minimal predictive information for future patients.

Threshold Excess (TE) demonstrated an inverse association with pain outcomes. Cross-validation suggested dose–response stability: each 1 mA above 2.0 mA predicted 6.4% less improvement (LOOCV β = −0.064 vs. apparent β = −0.063, 1.6% shrinkage; RMSE = 0.249 [fraction of percentage improvement; ≈24.9 percentage points]). Additional cross-validation details including k-fold validation and bootstrap confidence intervals are provided in Supplementary Materials (see, Supplementary Materials File S4).

### 3.9. Exploratory ROC-Derived Cut-Points (Hypothesis-Generating)

ROC analysis identified two candidate cut-points:

1. Proposed Ideal Target (≤2.0 mA, TE = 0):
  - Specificity: 75%;
  - Positive predictive value: 94.4%;
  - 6.1-fold increased odds of complete pain relief (*p* = 0.013).
2. Proposed Pragmatic Target (≤3.5 mA, TE ≤ 1.5):
  - Sensitivity: 76%;
  - Specificity: 75%;
  - Positive Predictive Value: 96.7%.

### 3.10. Exploratory Analysis: Robustness to Nerve Aggregation Strategy

To assess robustness to nerve-aggregation choices, we repeated analyses using the index nerve (highest pre-decompression threshold; *n* = 31, reflecting complete nerve-level linkage) and worst nerve (highest post-decompression threshold; *n* = 31). Core findings remained stable: TRR correlations 0.58–0.66 (*p* < 0.001) and TE slopes −0.053 to −0.063 (≈5.3–6.3% less improvement per 1 mA; 95% CIs exclude 0). Index-nerve aggregation provided numerically better cross-validated discrimination (LOOCV AUC 0.697 vs. 0.642 for mean aggregation), suggesting potential value in prioritizing the most affected baseline nerve. When restricting mean-aggregation to the same 31 patients, estimates were materially unchanged. Detailed results for all aggregation strategies are provided in Supplementary Materials (see, Supplementary Materials File S4).

## 4. Discussion

This pilot study compared different approaches for quantifying intraoperative MMG threshold changes and their associations with early pain outcomes following spinal decompression. Two candidate metrics anchored to a 2.0 mA reference threshold—Threshold Reduction Ratio (TRR) and Threshold Excess (TE)—showed substantially stronger associations with pain outcomes than percentage-based measures in this cohort (r = 0.656 vs. r = 0.397, representing 65% improved correlation). To our knowledge, this study provides the first quantification of dose–response relationships between post-decompression MMG threshold values and patient-reported outcomes, and suggests that measuring distance from a physiologic reference may better predict outcomes than arbitrary percentage improvement.

### 4.1. Interpretation of the 2.0 mA Threshold in Context

While only 42.5% (17/40) of patients achieved post-decompression thresholds ≤ 2.0 mA, 93% demonstrated clinical improvement and 90.5% achieved NPS MCID (≥2 points or ≥30%). This distinguishes between sufficiency for improvement versus optimization for superior outcomes.

Our data show a dose–response gradient in this cohort. Patients achieving ≤ 2.0 mA were associated with markedly superior outcomes: 76.5% complete pain relief versus 34.8% for those above 2.0 mA (OR = 6.1, *p* = 0.013). However, clinical improvement occurred across the full threshold range—even patients achieving only partial reduction showed measurable benefit.

This suggests 2.0 mA functions as an excellence target predicting exceptional outcomes rather than a necessary threshold for improvement. Our cohort’s mean post-decompression threshold of 3.26 ± 2.20 mA—above the 2.0 mA reference—occurred despite excellent aggregate outcomes (79% mean pain improvement, 90.5% MCID achievement).

The finding that further reduction below 2.0 mA showed no additional association with pain improvement (r = 0.094, *p* = 0.719) among the 17 patients achieving this threshold supports the concept of a functional sufficiency plateau, though this requires validation in larger cohorts.

Optimal achievable thresholds may vary by patient characteristics including age, chronicity, comorbidities, and baseline severity. This supports a personalized medicine framework where threshold targets are individualized rather than universal. A young patient with acute compression might be expected to achieve ≤2.0 mA reliably, while an elderly patient with chronic compression and comorbidities might have a more modest physiologic ceiling—perhaps 3–4 mA representing their optimal achievable outcome. Critical future work should develop and validate patient-specific threshold calculators that integrate these factors to provide individualized intraoperative targets. The finding that 52.2% of improving nerves achieved complete normalization demonstrates this threshold is frequently achievable, supporting its utility as an aspirational target while acknowledging patient-specific variation.

### 4.2. Comparison to Existing Literature

Our study advances prior descriptive work by addressing three critical gaps. Wessell et al. established that compressed nerves have elevated thresholds, decompression reduces these thresholds, and correlation exists with pain outcomes (*p* < 0.001). We quantified the strength (r = 0.656 for threshold-anchored metrics vs. r = 0.397 for percentage measures), characterized the functional form (linear: −6.3% pain improvement per mA above 2.0 mA, 95% CI −9.9% to −2.7%), and demonstrated that measuring distance from a reference threshold substantially outperforms arbitrary percentage improvement. Where Wessell demonstrated that correlation exists, we quantify its magnitude and shape.

The distinction also lies in the clinical question addressed: Wessell used a stimulation threshold of 2.0 mA to identify which pre-decompression nerves were pathologically compressed. We examined which post-decompression threshold values are associated with optimal outcomes—shifting from diagnosis to prognosis.

Limbrick and Wright examined EMG stimulation thresholds during lumbar discectomies, finding mean threshold reduction of 4.4 ± 4.0 mA [36]. However, their pre-decompression thresholds averaged 8.6 mA and post-decompression values only reduced to 4.2 mA—more than twice the 2.0 mA range expected for normal nerves. This may reflect EMG’s inherently higher baseline noise requiring larger signals for detection, or genuine incomplete decompression. Our MMG-based findings suggest achieving lower absolute post-decompression thresholds is associated with superior outcomes, raising questions about whether EMG’s higher baseline noise may obscure clinically relevant information.

Recent work by Sommer et al. examined MMG in minimally invasive spine procedures, finding varied utility across surgical approaches. Their observation that MIS-TLIF showed minimal threshold changes because facetectomy (a decompressive maneuver) precedes nerve identification aligns with our limited TLIF experience. This highlights that procedural timing affects MMG utility: procedures allowing early nerve access before decompression (foraminotomy, far lateral discectomy) enable true baseline measurement, while procedures with obligate early decompression require alternative strategies. Table 5 summarizes key prior studies in MMG and EMG threshold assessment for spinal nerve evaluation, contextualizing the current work’s contributions.

### 4.3. Quantifying the MMG-Outcome Relationship: Novel Contributions

This study’s contributions lie in three specific advances beyond prior descriptive work:

1. *Comparative Framework Analysis:* We systematically compared three quantification approaches—percentage change, Threshold Reduction Ratio (TRR), and Threshold Excess (TE)—demonstrating that threshold-anchored metrics outperform percentage-based measures by 65% (r = 0.656 vs. r = 0.397). This methodological finding suggests that how we quantify nerve recovery matters substantially for outcome prediction.
2. *Dose–Response Characterization:* We characterized the functional form and strength of the threshold-outcome association: each 1 mA of post-decompression threshold remaining above 2.0 mA was associated with 6.3% less pain improvement (β = −0.063, 95% CI −0.099 to −0.027, *p* = 0.001). This linear relationship, if replicated in future cohorts, could inform trial design and intraoperative decision frameworks.
3. *Outcome Stratification:* We observed outcome gradients across threshold categories. Patients achieving ≤ 2.0 mA were associated with 76.5% complete pain relief versus 34.8% above 2.0 mA (OR = 6.1, *p* = 0.013), with progressive deterioration across tertiles. This stratification transforms MMG from a binary assessment of improvement to a continuous prognostic tool scaled by achieved threshold values.

### 4.4. Toward Personalized Surgical Decision-Making

If validated prospectively, threshold-anchored MMG metrics could enable a shift from standardized decompression protocols to personalized surgical decision-making. Rather than applying uniform decompression extent across all patients, surgeons could tailor intervention intensity based on individual nerve responses. A patient achieving rapid threshold normalization (e.g., 8→2 mA after limited decompression) may require less extensive intervention, while another showing persistent elevation (e.g., 10→7 mA) might benefit from more extensive decompression—provided anatomic constraints and complication risk permit. This represents data-driven, individualized surgical care where each patient’s decompression extent is optimized for their specific nerve physiology. The complementary TRR/TE framework may offer both mechanistic understanding (TRR: what proportion of elevation was eliminated) and operational benchmarking (TE: how much excess remains relative to reference).

The sole case of concordant non-improvement (unchanged MMG and pain) despite adequate intraoperative visual assessment suggests potential clinical value, though causality cannot be inferred from a single case. However, whether threshold persistence indicates inadequate decompression versus irreversible neural damage requires further investigation through controlled trials.

The technology’s practical advantages include surgeon-driven operation without dedicated neurophysiology technicians, reducing cost and staffing requirements. Setup requires <5 min, and threshold testing adds <2 min per nerve. In our experience, MMG provided clear, noise-free signals in all cases, contrasting with EMG’s susceptibility to electrical interference.

### 4.5. Candidate Thresholds for Future Investigation

We observed associations between post-decompression thresholds and outcomes. Whether targeting these thresholds improves outcomes requires prospective testing.

In this cohort, patients achieving patient-level mean post-decompression thresholds ≤ 2.0 mA (TE = 0) were associated with 76.5% complete pain relief versus 34.8% for those above 2.0 mA. The observed linear relationship suggests each 1 mA above 2.0 was associated with 6.3% less pain improvement (95% CI −9.9% to −2.7%).

These findings come from a highly selected population with 93% improvement rates and require validation in typical surgical cohorts. If confirmed through prospective trials, such associations could inform intraoperative decision frameworks while balancing decompression adequacy against procedural risk.

### 4.6. Patient-Specific Threshold Variation: Toward Individualized Targets

A critical limitation of the current study is that we evaluated a single reference threshold (2.0 mA) across all patients. While this population-level approach enabled initial signal detection, true personalized medicine requires patient-specific threshold targets. Several factors likely modulate optimal achievable thresholds:

*Age and Neurophysiology:* Older patients may have baseline age-related changes in nerve excitability, independent of compression. A 70-year-old achieving 3.0 mA post-decompression may represent complete physiologic recovery for their age, while the same threshold in a 35-year-old might indicate residual compression.

*Chronicity and Nerve Damage:* Patients with chronic compression (>2 years) may have irreversible axonal damage limiting recovery potential. These patients might achieve optimal outcomes at 3–4 mA if their nerves cannot fully recover, regardless of decompression adequacy.

*Comorbidities:* Diabetes, peripheral neuropathy, and inflammatory conditions affect baseline nerve function. These patients may have elevated “normal” thresholds, requiring adjusted targets.

*Baseline Severity:* Patients with extreme baseline thresholds (>15 mA) might show excellent outcomes with reduction to 4–5 mA, while those starting at 5 mA might need to reach 2.0 mA for similar relief.

Future research should develop and validate patient-specific threshold models. Machine learning approaches could integrate preoperative variables (demographics, symptom duration, imaging findings, baseline thresholds) to generate individualized threshold targets. Such models would enable truly personalized surgical decision-making, where a 75-year-old diabetic with 5-year symptom duration receives a different target threshold than a 40-year-old with 3-month symptoms—both optimized for their specific physiology.

Until such models are validated, the 2.0 mA threshold should be interpreted as an aspirational target associated with excellent outcomes in our cohort, acknowledging that patient-specific factors may modulate optimal achievable values.

### 4.7. Study Limitations

*Metric Development and Internal Validation:* While TRR and TE were developed on this dataset, rigorous leave-one-out cross-validation demonstrated excellent generalization for TRR (9.3% shrinkage) and stable dose–response coefficients for TE (1.6% shrinkage). These findings indicate threshold-anchored metrics capture genuine predictive signal rather than statistical artifacts. Nevertheless, external validation in independent cohorts from multiple centers remains essential to confirm these findings and establish definitive clinical utility across diverse patient populations and practice settings.

*Study Design:* The observational, non-randomized design demonstrates associations between MMG thresholds and outcomes but cannot establish causation. Lower post-decompression thresholds may result from more complete decompression (causal), may simply identify patients with more reversible pathology (selection), or both may be caused by less severe baseline compression. Only randomized trials comparing MMG-guided surgery to standard care can determine whether incorporating threshold feedback into surgical decision-making improves patient outcomes. Furthermore, we cannot determine whether pursuing lower thresholds in unfavorable anatomy might increase complication risk without benefit.

*Analytical Approach:* The approved IRB protocol specified no statistical analyses would be performed. During manuscript development, we conducted statistical testing to quantify associations and generate hypotheses. While appropriate for hypothesis-generating studies, our analytical approach was not pre-registered. Multiple comparisons and iterative analysis increase risk of Type I error. Future studies should pre-specify metrics, thresholds, and statistical plans to reduce researcher degrees of freedom.

*Cohort Selection:* We intentionally studied patients with high expected clinical success rates (93% improvement) to establish initial signal before introducing patient heterogeneity that may modulate the relationship. While appropriate for hypothesis generation, this limits generalizability to typical surgical populations. Patients with comorbidities (diabetes, peripheral neuropathy, significant psychological disturbance) were excluded, and all procedures were performed by experienced fellowship-trained spine surgeons. We anticipate optimal thresholds may require patient-specific refinement based on comorbidities, chronicity, baseline severity, and anatomical factors.

*Short-Term Follow-up:* Six-week outcomes capture only early recovery. While early pain improvement correlates with long-term outcomes, the ultimate measure of decompression adequacy is revision surgery rates. Longer follow-up (minimum 2 years) is required to correlate MMG findings with reoperation rates—the definitive clinical endpoint.

*Sample Size and Exploratory Findings:* While appropriate for pilot methodology (*n* = 42), subgroup analyses remain exploratory. Stratified analyses by TRR and TE tertiles included only 8–17 patients per group, limiting precision of effect estimates. The finding that 2.0 mA represents a sufficiency plateau derives from only 17 patients and requires validation in larger cohorts.

*Threshold Reference Validity:* While the 1–2 mA range for normal nerve function was pre-specified in our IRB protocol based on published MMG literature, we cannot definitively distinguish whether observed thresholds represent physiologic phenomena versus equipment-specific characteristics. The threshold may require patient-specific calibration based on comorbidities, chronicity, and baseline severity. Our highly selected cohort may have enabled correlations that wouldn’t generalize to typical surgical populations.

*Procedural Limitations:* For surgical approaches where decompression precedes nerve identification (e.g., MIS-TLIF with facetectomy before nerve access), obtaining true pre-decompression baselines may be impossible.

## 5. Conclusions

In this exploratory pilot study, threshold-anchored metrics (TRR: r = 0.656; TE: r = −0.500) showed stronger correlations with pain outcomes than percentage-based measures (r = 0.397), representing 65% improved correlation in this cohort. We observed a dose–response relationship: each mA above 2.0 mA was associated with 6.3% less pain improvement (95% CI −9.9% to −2.7%). In this preliminary study, patients achieving ≤ 2.0 mA were associated with 6.1-fold increased odds of complete pain relief (76.5% vs. 34.8%, *p* = 0.013).

The key conceptual contribution is demonstrating that measuring how far a nerve moves toward a clinically referenced threshold (2.0 mA) may better predict clinical outcomes than calculating percentage change from arbitrary baselines. Our findings suggest a dose–response gradient between post-decompression thresholds and clinical outcomes, with 2.0 mA functioning as an excellence target associated with superior outcomes while clinical improvement occurs along a continuum. Internal cross-validation suggested internal stability in this cohort, with TRR demonstrating minimal shrinkage (9.3%) and TE showing stable dose–response relationships.

These exploratory findings provide preliminary evidence and effect-size estimates for future prospective studies. However, the specific metric formulations (TRR and TE), numerical effect estimates (r = 0.656, 6.3% per mA), and threshold values (2.0 mA) require external validation in independent cohorts before clinical implementation. Until prospectively validated in multicenter trials, these metrics should be considered investigational tools for research purposes rather than clinical decision-making.

If validated prospectively, these metrics could enable surgeons to tailor decompression extent to individual nerve physiology rather than relying on uniform anatomical criteria, advancing personalized approaches to spine surgery. These preliminary findings provide effect-size estimates and proof-of-concept for a testable framework enabling multicenter randomized trials to determine whether MMG-guided decompression reduces revision surgery rates compared to standard care. Critical next steps include: (1) external validation of TRR and TE in independent cohorts to assess generalizability, (2) prospective observational studies to determine whether real-time MMG feedback modifies surgical decision-making, (3) development of patient-specific threshold calculators that integrate demographic, clinical, and physiologic factors to individualize the 2.0 mA reference threshold for each patient’s optimal achievable outcome, and (4) ultimately, randomized trials to establish whether MMG-guided surgery improves outcomes. Only through this systematic validation pathway can threshold-anchored MMG metrics transition from exploratory research tools to evidence-based clinical decision aids.

## Figures and Tables

**Table 1:** Baseline Characteristics (*n* = 42).

Characteristic	Value
Procedure type	
Lumbar decompression, n (%)	32 (76)
Cervical decompression, n (%)	10 (24)
Surgical levels	
Single level, n (%)	24 (57)
Multiple levels, n (%)	18 (43)
Nerves tested	
Total nerves, n	112
Nerves per patient, mean (range)	2.7 (1–6)
Baseline radicular pain	
Numeric Pain Scale, mean ± SD	6.8 ± 2.4
Patients with NPS > 0, n (%)	40 (95.2)

**Table 2:** Outcomes Stratified by Threshold Reduction Ratio.

TRR Category	n	Mean Pain Improvement (95% CI)	Complete Relief Rate (95% CI)
TRR = 1.0 (complete normalization)	17	92.5% (84.0–100%)	76.5% (52.7–90.4%)
0.6 ≤ TRR < 1.0 (substantial reduction)	15	81.3% (71.4–91.2%)	40.0% (19.8–64.3%)
TRR < 0.6 (partial reduction)	8	53.1% (22.3–84.0%)	25.0% (7.1–59.1%)

**Table 3:** Outcomes Stratified by Threshold Excess.

TE Category	n	Mean Pain Improvement (95% CI)	Complete Relief Rate (95% CI)
0.0 ≤ TE < 0.5 mA (at/near reference)	17	92.5% (84.0–100%)	76.5% (52.7–90.4%)
0.5 ≤ TE < 2.5 mA (moderate excess)	14	80.5% (69.1–91.9%)	42.9% (21.4–67.4%)
TE ≥ 2.5 mA (substantial excess)	9	57.5% (30.1–84.9%)	22.2% (6.3–54.7%)

**Table 4:** Internal Cross-Validation Performance.

Metric	Apparent r (95% CI)	Cross-Validated r (LOOCV)	Shrinkage
%MMG Change	0.397 (0.097–0.630)	0.201	49.3%
Threshold Reduction Ratio	0.656 (0.426–0.807)	0.597	9.3%
Threshold Excess	−0.500 (−0.702 to −0.223)	–	*
Metric	Apparent R^2^	CV R^2^	R^2^ Loss
%MMG Change	15.7%	4.0%	74.5%
Threshold Reduction Ratio	43.4%	35.6%	18.0%
Threshold Excess	25.0%	12.6%	49.6%

LOOCV = leave-one-out cross-validation; CV = cross-validated. * Note: For TE, predictive accuracy is better summarized by LOOCV RMSE and R^2^ (not correlating predictions with outcomes): RMSE = 0.249 (fraction of percentage improvement; ≈24.9 percentage points), R^2^_CV = 0.126; the dose–response coefficient showed negligible shrinkage (apparent β = −0.063; LOOCV β = −0.063).

**Table 5:** Comparison of Key MMG and EMG Studies in Spinal Nerve Assessment.

Study	Procedure (s)	Measurement Modality	Key Findings
Holland et al., 1998 [23]	Lumbar decompression	EMG threshold testing	Chronically compressed nerve roots require higher electrical stimulus intensities for activation
Wessell et al., 2016 [24]	Lumbar/cervical decompression	MMG	90% had elevated pre-decompression thresholds (≥2.0 mA); 98% showed ≥1 mA reduction; mean post-decompression 2.04 ± 2.22 mA; VAS pain improved 6.8→1.1 (*p* < 0.001)
Limbrick & Wright 2005 [36]	Lumbar microdiscectomy	EMG threshold testing	Mean threshold reduction 4.4 ± 4.0 mA (8.6→4.2 mA)
Guerrero et al., 2023 [20]	Peripheral nerve decompression	MMG	80% had detectable MMG signals with significant threshold improvement (1.59 ± 0.19 mA → 0.47 ± 0.03 mA, *p* = 0.0002)
Javeed et al., 2023 [21]	Peripheral nerve decompression	MMG	MMG thresholds correlated with CMAP and clinical outcomes (PROMIS-PF, ODI, *p* < 0.05)
Sommer et al., 2025 [25]	Cervical foraminotomy, far lateral discectomy, MIS-TLIF	MMG	Mean threshold reductions: 3.4 mA (cervical foraminotomy), 4.4 mA (far lateral discectomy); minimal change in MIS-TLIF due to early decompression before nerve identification
Current study	Lumbar/cervical decompression	MMG	Threshold-anchored metrics (TRR, TE) outperformed percentage-based measures (r = 0.656 vs. r = 0.397); linear dose–response (6.3% less improvement per mA above 2.0 mA); patients achieving ≤ 2.0 mA: 76.5% complete relief vs. 34.8% (OR = 6.1)

## Data Availability

The de-identified original dataset, original contributions presented in this study, and analysis code supporting the findings of this study are provided in the article/Supplementary Materials. Further inquiries can be directed to the corresponding author. All personally identifiable or time-stamped intraoperative data have been removed to comply with institutional privacy requirements under IRB #14745.

## Supplementary Materials

The following supporting information can be downloaded at: https://www.mdpi.com/article/10.3390/jpm15120564/s1. File S1: MMG_Datafile.xlsx—patient-level and nerve-level variables (*n* = 42 patients, 112 nerves); File S2: MMG_Submission_Supplement.xlsx—aggregated summary tables; File S3: mmg_comprehensive_supplement.py—reproducible analysis code; File S4: Supplementary_Materials_Statistical_Validation.docx—Statistical Validation and Robustness Analyses (detailed cross-validation results, bootstrap confidence intervals, and nerve aggregation strategy comparisons).
