# Supplementary material for: Threshold-Anchored Mechanomyography Metrics for Patient Stratification in Spinal Decompression: Associations with Early Pain Outcomes
Source: J Pers Med. 2025 Nov 21;15(12):564. doi: 10.3390/jpm15120564 (PMC12734088; doi:10.3390/jpm15120564)
Supplement: Supplementary file 1 [file jpm-15-00564-s001.zip › Supplementary File S4-Statistical_Validation.pdf]

Journal of
Personalized Medicine

MDPI

Internal Cross-Validation Analysis of MMG Quantification Metrics

Table S1: Correlation Performance.

Metric	Apparent Correlation r (95% CI)	Cross-Validated Correlation r (LOOCV)	Shrinkage (%)
%MMG Change	0.397(0.097-0.630) $p = 0.011$	0.201	49.3%
Threshold Reduction Ratio (TRR)	0.656(0.426-0.807) $p < 0.001$	0.592	9.3%
Threshold Excess (TE)	-0.500(-0.702 to -0.223) $p = 0.001$	–	*

* Note: For TE, predictive accuracy is better summarized by LOOCV RMSE and R^2 (not correlating predictions with outcomes): RMSE = 0.249, R^2_CV = 0.126; the dose–response coefficient showed negligible shrinkage (apparent β = −0.063; LOOCV β = −0.063).

Table S2. Variance Explained (R^2).

Metric	Apparent R^2 (%)	CV R^2 (%)	R^2 Loss (%)
%MMG Change	15.7	4.0	99.3
Threshold Reduction Ratio (TRR)	43.1	35.6	18.0
Threshold Excess (TE)	25.0	12.6	70.6

Table S3. Bootstrap Cis.

Parameter	Wald 95% CI	Bootstrap 95% CI
Threshold Excess Slope (β per mA)	-0.98 to -0.28	-0.133 to -0.021 (percentile)
Threshold Excess OR (complete reflief)	0.36-0.98	0.20-0.87 (percentile)

Table S4. Relative Performance Comparison.

Comparison	Apparent (Full Data)	Cross-Validated (LOOCV)	Statistical Test
**TRR vs %MMG**	0.656 vs 0.494	0.592 vs 0.201	Steiger's Z = 2.09, p = 0.037

Relative improvement in r: Apparent = 33.0%; Cross-validated = 197% (Computed as $(r_{\mathrm{TRR}} - r_{\%\Delta\mathrm{MMG}})/r_{\%\Delta\mathrm{MMG}}$).

Interpretation: The difference in apparent correlations is statistically significant (*Steiger's* Z = 2.09, $p = 0.037$) when both correlations are computed on the same n=38 patients. TRR explains nearly 1.8× the variance (R^2 = 43.4% vs 24.4%) and shows vastly superior generalizability under cross-validation (r = 0.592 vs 0.201; 197% relative improvement; 9.3% vs 49.3% shrinkage).

Table S5. K-Fold Cross-Validation Confirmation.

Metric	5-Fold CV Mean r ± SD	10-Fold CV Mean r ± SD	LOOCV r
%MMG Change	0.390 ± 0.408	0.303 ± 0.541	0.201
Threshold Reduction Ratio (TRR)	0.515 ± 0.447	0.389 ± 0.473	0.592
Threshold Excess (TE)	0.502 ± 0.403	0.450 ± 0.381	0.355

NOTES

Abbreviations: LOOCV, leave-one-out cross-validation; CV, cross-validated; TRR, Threshold Reduction Ratio; TE, Threshold Excess; %MMG, percentage change in mechanomyography threshold; CI, confidence interval; SD, standard deviation

***Note on Cross-Validation:** For continuous outcomes, we assess predictive accuracy via LOOCV RMSE and R^2_CV. For TE: RMSE = 0.249, R^2_CV = 0.126. The dose–response coefficient (β) showed negligible shrinkage (apparent β = −0.063 vs LOOCV β = −0.063), confirming stability.

Methods: Leave-one-out cross-validation was performed for all 40 patients with baseline pain >0. For each patient, linear regression models were trained on 39 patients and tested on the held-out patient. This process was repeated 40 times. Cross-validated correlations represent the correlation between actual outcomes and predictions from held-out test sets. Shrinkage represents the percentage decrease from apparent to cross-validated performance; values <20% indicate robust generalization to new patients.

Interpretation Guidelines: - **Low shrinkage (<20%):** Excellent generalization, clinically useful predictor - **Moderate shrinkage (20-50%):** Acceptable but warrants external validation - **High shrinkage (>50%):** Poor generalization, minimal predictive value

Key Finding: TRR demonstrates excellent generalization (9.3% shrinkage) suitable for clinical decision-making pending external validation, while percentage-based metrics show poor predictive validity (49.3% shrinkage) when properly validated.

Robustness to Nerve Aggregation Strategy

Table S6. Robustness to Nerve Aggregation Strategy.

Aggregation Method	n	r(TRR, pain)	r(TE, pain)	TE Slope (per mA)	OR per mA TE (complete relief)	LOOCV AUC
Mean (all nerves)	40	0.656***	-0.500**	-0.063**	0.60*	0.642
Pre-Max†	31	0.653***	-0.540**	-0.054**	0.70*	0.697
Post-Max‡	31	0.578***	-0.521**	-0.053**	0.72*	0.676

*p<0.05; **p<0.01; ***p<0.001

Abbreviations: TRR, Threshold Reduction Ratio; TE, Threshold Excess; OR, odds ratio; LOOCV, leave-one-out cross-validation; AUC, area under curve

† Pre-Max = examine only highest pre-decompression threshold per patient (proxy for most symptomatic)

‡ Post-Max = examine only highest post-decompression threshold per patient (greatest residual pathology)

Note: n=31 reflects availability of complete nerve-level linkage data. Mean-aggregation analyses used n=40; restricting mean-aggregation to n=31 yielded materially similar estimates.
